# Mercantilist and protectionist shocks on innovation, growth, and economic policy in European regions

**DOI:** 10.1007/s00191-025-00921-w

**Published:** 2026-01-19

**Authors:** Philip McCann, Raquel Ortega-Argilés, Mark Thissen, Ming-Wei Hsu

**Affiliations:** 1 The Productivity Institute, Alliance Manchester Business School, University of Manchester, Manchester, UK; 2 PBL Netherlands, The Hague, The Netherlands

**Keywords:** Innovation, Trade, Global value chains, Regions, Europe, R11, O47, O33, F14

## Abstract

We assess the potential innovation and growth implications on European regions of the rapid global shifts in political economy towards mercantilism, tariffs, and protectionism. The new trade shocks have the potential to reshape the geography of European innovation, and we examine the likely global value-chain implications on regional innovation in both STI-driven and DUI-driven regions using a unique EU-wide regional input–output database. The analyses undertaken here using the EU *EUREGIO*-FIGARO datasets allow us to incorporate not only the direct demand-transmission effects of international trade on different EU regions, but also the full trade-in-value-added logic, which also includes the indirect effects of GVCs spanning EU regions and non-EU countries. We demonstrate that both STI-driven and non-STI/DUI-driven regions are exposed in different ways to these trade shocks, and that Europe’s territorial innovation, growth, and development challenges are likely to become even more complicated in today’s global political context, depending on where regions are positioned in global value chains.

## Introduction

This paper aims to reassess the potential innovation and growth implications across different types of STI- and DUI-driven European regions in the context of the current, rapid global shifts in the political economy. STI (science-technology-innovation)-driven regions are the types of regions innovation scholars have been most focused on in during the last three decades, but in more recent years there has been a rapidly growing literature discussing the DUI (doing-using-interacting)-driven modes of innovation and the types of regions characterized primarily by these types of innovation processes (Jensen et al. 2007; Isaksen and Karlsen 2016; Thomä 2017; Aslesen and Pettersen 2017; Alhusen and Bennat 2021; Alhusen et al. 2021; Friedrich and Sternberg 2025; Haus-Reve et al. 2023; Najda-Janoszka 2025). Rather than innovation being driven primarily by science, technology, and R&D, in DUI-driven regions, tacit know-how and experience-led innovation takes place primarily by a variety of different routes, including changes in: production systems, organizational templates, skills augmenting and re-skilling programs, product redesign and re-engineering, supply-chain reconfiguration, capital and machinery enhancements. In these types of DUI-driven regional innovation systems, the roles of local business networks and communities of practice are critical in fostering key knowledge exchanges for learning (Friedrich and Sternberg 2025), and innovation is typically incremental, but can sometimes be fundamental and radical.

The supply chains and business networks in which firms are situated are essential mediators of innovation in DUI systems, and major disruptions to these systems can threaten their flourishing or survival. If local supply chains are part of broader global value chains, then local innovation systems are exposed to global shocks. In particular, the advent of the Trump administration in the USA has ushered in a rapid and profound shift in the economics of the global political economy from one based on principles of specialization and comparative advantage to one increasingly driven by mercantilist and tariff-induced protectionist thinking. In this new framing, scale and trade balances are perceived of as being central to political decision-making, a logic known as ‘geonomics’ (Clayton et al. 2025; Tett 2025a), and strategic, political and diplomatic decisions are based on these principles rather than the neoliberal logic of trade and global value chains (Tett 2025b) which has underpinned the geography of innovation ‘pipelines’ (Fitjar and Rodriguez-Pose 2014) for most of the last four decades. Many aspects of the old global economic order, which has existed in various guises since the Bretton Woods Agreement of 1944, have largely disappeared, and many people in Europe are rethinking the roles that the EU plays in the global financial, investment, savings, consumption, and fiscal balances (MGI 2025; Wolf 2025a). Indeed, global trade shocks are likely to be keenly felt in the EU. Trade in goods between EU countries, namely intra-EU trade, is some 56% higher than the extra-EU trade level recorded for exports leaving the EU to non-EU member countries (Eurostat 2024). However, the trade surpluses generated by trade between EU member states and other non-EU countries have been increasing in recent years, and this is the case for both the Euro-area countries (Eurostat 2025a) and the wider EU, including non-Euro countries (Eurostat 2025b; EIB 2025). Importantly, the US and China are the largest external markets for EU exports, and while the EU runs a large trade deficit with China (EIB 2025), the EU displays a large trade surplus with the USA (EIB 2025), opening it up to the threat of tariffs from the Trump administration.

As we will show in this paper, these tariffs threaten the innovation potential of different European regions in different ways because their local economies are differently vulnerable to tariff-related and protectionist shocks. This is because the innovation, growth, and development implications on EU regions of any particular tariffs on EU exports and imports, depend on the interactions between not only the affected internationally traded sectors, but also the interactions between the tariffed sectors and all other tariffed and non-tariffed international and domestic sectors. Innovation thrives in buoyant local economies because entrepreneurs and firms are more willing to take risks when economies are strong, and financiers are also more willing to back those risks in more buoyant economies. However, observations of the levels of tariffs and the sectors on which they are levied do not provide a comprehensive assessment of the likely overall local growth and development implications of the tariffs, because the impacts of tariffs on one sector in one region are passed on through to other sectors in the same region and in other regions in different ways, depending on the regional and interregional economic structures.

This is important because innovation cannot be treated in isolation, but as part of a wider restructuring of the trade relationships underpinning the global economic system, and in which Europe potentially needs to play a central role in order to maintain the underpinnings of our liberal economies. These are issues which were initially picked up by the recent reports produced by two former Prime Ministers of Italy, namely Mario Draghi (Draghi 2024a, b) and Enrico Letta (Letta 2024), which focused on the weak growth problems of the EU economies and the need to stimulate innovation. After decades of deregulation and harmonization, the EU today has fewer internal barriers to trade than ever. However, the EU Single Market remains fragmented, with hundreds of trade and non-trade barriers remaining within the EU, such that, in this era of trade wars, Brussels is prioritizing the enhancement and deepening of the EU Single Market (Moens and Hancock 2025). However, the scale of the changes wrought by the Trump administration goes far beyond what was envisaged in either the Draghi or Letta reports, and requires a wholesale reconsideration of the role played by trade shocks in impacting on EU regional innovation, growth, and development processes. We investigate these issues in the context both of European regions characterized primarily by science and technology innovation (STI) modes of development and also regions characterized primarily by doing, using, and interacting (DUI) modes of innovation**,** and we will demonstrate that all types of EU regions are exposed trade-related risk and uncertainty, depending on where they are positioned in global value chains (GVCs).

To analyze the effect of tariff-induced demand falls from the US or from China on European regional GDP via value chains, we use input–output analysis applied to the regional extensions of the EU FIGARO data system, namely the EUREGIO datasets. These datasets provide the full input–output and interregional trade structures of 242 NUTS2 regions across 64 different sectors covering all 27 EU member states, plus the UK and Norway, and all linked to the EU’s largest external trading partner countries, as well as the rest of the world combined. The fact that these data have been regionalized means that they explicitly allow us to examine how changes in trade and demand in different parts of the world impact on different EU regions in different ways in terms of the implications for the destination of outputs, the origin of inputs, the global value chains (GVCs) embedded in different EU and non-EU countries, and the risks around these trade and value-adding patterns.

The construction of these datasets by Eurostat is explicitly intended to facilitate analyses of the EU’s experience and performance in terms of economic globalization, economic and social performance, and environmental sustainability (Remond-Tiedrez and Rueda-Cantuche 2019). Indeed, as the European Commission itself states, “*The FIGARO tables are linked to the Commission priorities: ‘A stronger EU in the world’ and ‘European green deal’ by providing policy makers with necessary data for models and different analyses (e.g., jobs, environment-related issues,* *etc.)*”^1^. Our approach, using the EUREGIO datasets set at the regional rather than the country level, is especially pertinent in the context of this discussion, given that national tables cannot identify regional (Giammetti et al. 2022) or welfare inequality (Campos et al. 2023) effects within countries, and the indirect demand effects of global value chains (GVCs) will become increasingly important as global trade fragmentation, near-shoring or ‘friend-shoring and regionalization increases (Conteduca et al. 2025). Using an earlier generation of the EUREGIO datasets, Giammeti et al. (2022) found that regional production networks within the EU became increasingly fragmented between 2000 and 2010, although the trend towards production fragmentation outside the EU was faster (Giammeti et al. 2022). This was some 15 years before the recent global mercantilist trade shocks. The analysis of these new-generation EUREGIO datasets therefore allows us to discuss which types of EU regions are most likely to be affected by these recent, quite different tariff-related impacts and what the regional innovation and implications of these new tariff systems are likely to be. We demonstrate that all types of EU regions in terms of their innovation characteristics, whether STI-driven regions or non-STI/DUI-driven regions, face significant trade-related risks and uncertainty, and that there are no simple STI/DUI region or leading/lagging region dichotomous patterns to these risks. It depends on the specific positioning of individual regions within global value chains (GVCs). We conclude by discussing the implications of these results for the growing concerns for Europe’s economic future raised by the reports of Mario Draghi (Draghi 2024a, b), Enrico Letta (Letta 2024), and mission-led policies.

The results of this paper are novel in that the implications of global mercantilism for the innovation risks faced by different types of EU regions have not been laid out before in any systematic manner, and indeed, these issues were almost entirely ignored by both Draghi and Letta (McCann and Stierna 2025). What our research allows us to demonstrate is that there are large geographical and structural differences in terms of the regional shocks associated with different types of mercantilist shocks, depending on the source of the shocks. In particular, as well as impacting differently on leading and lagging EU regions, these shocks also impact differently on different innovation-types of EU regions, both STI-driven and DUI-driven regions, thereby raising important questions regarding the future design and delivery of EU regional innovation policies.

The rest of the paper is organized as follows. In the next section, we discuss the background to the recent rapidly shifting links between innovation, trade, and growth as articulated in European policy debates, and the potentially profound shocks to these links due to mercantilist and protectionist approaches now being deployed in the global economy. In the third section, we use the regional extensions of the EU FIGARO datasets in order to examine the demand side, supply side, and global value chain (GVC) economic risk exposure of European regions to tariff barriers and trade wars, and we also extend this analysis to include the additional uncertainty regarding the source of the shocks. This provides us with a mapping of the likely European economic geography of local adverse demand shocks due to trade disruption. These results are then translated into the likely impacts according to different types of STI-driven and non-STI/DUI-driven regions, based on their innovation characteristics. We see that the results differ markedly between different types of regional innovation features, both in terms of the average levels and also the potential variation in their risk exposure, depending on where regions are positioned in global value chains (GVCs). In the fourth section, we discuss in detail the extent to which high-level EU policy narratives, as encapsulated in the Draghi (2024a, b) and Letta (2024) reports, fail to reflect the fundamental underlying regime changes in the European growth model from one of strong-growth-and-convergence to weak-growth-and-divergence. Consequently, it is argued that these high-level policy narratives not only fail to grasp many aspects of the growth-related challenges that the European economies face, but also that in the context of the emerging trade-related risks and uncertainties facing different innovation-types of EU regions, some of the likely political responses have the power to exacerbate these risks and uncertainties, thereby jeopardizing European efforts towards open strategic autonomy. Finally, the fifth section provides some brief conclusions.

## Background to the shifting links between trade, innovation, and growth

In his two-part report of 2024, Mario Draghi (Draghi 2024a, b) raised major concerns regarding the long-term competitiveness of Europe in the newly emerging world order, which involves major shifts in the patterns, mechanisms, and rules of trade, energy, and defense. He argued that European policy and regulatory responses to these challenges must focus on three major arenas, namely: innovation in advanced technologies, decarbonization, and the need for enhanced economic, technological, and military security (Draghi 2024a). Draghi argued for regulatory transformations in these arenas, focused on building EU-wide scale and integration in those arenas essential for matching the progress already being made in the USA and China. A parallel report by Enrico Letta (Letta 2024) also raised many of these concerns in the specific context of the workings of the EU Single Market, but while the latter report (Letta 2024) discussed some of the EU’s regional and place-based features, the former report (Draghi 2024a, b) made almost no mention of these features. In particular, both the Draghi report (Draghi 2024a, b) and the Letta report (Letta 2024) discussed EU growth and innovation in an entirely space-blind manner akin to the EU growth trajectories of the pre-crisis era (Barro and Sala-i-Martin 1995) when EU-wide growth and EU-wide interregional convergence were essentially two sides of the same coin, in essence, the same thing. As we discuss in more detail later, no mention in either report was made of the profound switch since the 2008 global financial crisis from a regime characterized by EU-wide interregional convergence to one characterized by EU-wide interregional divergence, in which many regions are increasingly being ‘left behind’, and resulting in a ‘geography of discontent’ (McCann and Ortega-Argilés 2021), which has the potential to undermine and thwart any EU-wide initiatives proposed by Draghi or Letta.

In the months since these reports first appeared, however, the whole global political economy of trade, defense, and sustainability has been thrown into turmoil by the *realpolitik* of the Trump administration. The thinking of the Trump administration has led to rapid and profound shifts in the global political economy of trade from a logic of economic engagement based on comparative advantage principles to a logic that is based on mercantilism, in which trade surpluses and deficits become central to political decision-making. In terms of innovation and growth, this cements a dramatic political economy shift away from a logic based on specialization to one based on scale and absolute advantage, and the US combination of economic leverage, military might, and digital-tech dominance will have profound economic and political implications for Europe, as Draghi warned, but not necessarily in the manner that Draghi envisaged. Indeed, no one foresaw the nature, speed, and scale of the shifts currently underway, in which many aspects of the post-war settlement are being fundamentally rewritten. In particular, the use of tariffs as an economic weapon was typically understood as something of the past, and a sanction to which recourse was only made in order to sanction unlawful state behavior. Now trade tariffs and technological restrictions are being reinvented by the Trump administration as a mainstream economic policy tool (Tett 2025a).

These realpolitik shifts have the potential to rewrite the geography of innovation, growth, and development across EU regions, depending both on the patterns of the trade shocks and also the EU’s responses. In academic arenas, the regional economic shifts from frameworks based on comparative advantage to ones increasingly driven by mercantilist thinking were already to some extent underway due to the literatures on competitive advantage, new economic geography and the urban economics of large cities, which ushered in indirectly related arguments regarding the centrality in the modern economy of scale-related absolute advantage, rather than comparative advantage (Porter 1990; Krugman 1991). However, the mercantilist and protectionist thinking of the Trump administration goes much further than pure scale arguments, emphasizing the relationships between trade balances and political bargaining, and using scale as the primary economic arbiter of the enforcement of a trade balance logic.

The mercantilist–protectionist logic underpinning the USA’s rapid movement towards tariffs is outlined by Stephen Miran, the Chair of the US President’s Council of Economic Advisors (Miran 2024). The focus of Miran’s argument is the so-called ‘Tiffin World’, named after the 1960 s Belgian economist Robert Tiffin, who posited that global reserve assets, such as the US dollar or US Treasuries, are a form of global money supply essential for facilitating global trade. The demand for these assets depends on global trade, and not on the specific trade position of the reserve asset issuer, namely the USA (Tiffin 1960). As such, on this argument, the US dollar typically tends to be systematically overvalued, leading to rapid declines in domestic US manufacturing (Miran 2024), the impacts of which are most adversely borne by the interior heartland regions.

In economic terms, this argument underpins the current tariff implementation logic of the USA, but this view is a only partial view, in that the implications of trade positions for US manufacturing are a natural outcome of the various balances between US, Chinese, European and global consumer expenditure, investment, savings and trade (MGI 2025; Wolf 2025a), in a setting where the US enjoys consistently greater financial inflows at lower risk premiums than other countries, due to the global reserve currency status of the US dollar, the reserve asset status of dollar-denominated US sovereigns, and the extent to which the dollar (Wolf 2025b). The US economy has largely outperformed most other OECD economies in the post-2008 crisis era, so rather than pure economics, the mercantilist–protectionist shift is likely to be primarily about power and security (Foroohar 2025), issues also raised as concerns by Draghi (2024a, b) in the European context. The current political logic of the US approach is underpinned both by the political power built on a voting base suffering from the adverse distributional implications of globalization on so-called ‘left-behind’ regions, and also concerns regarding the loss of advantages in key technologies essential for military purposes. However, there are doubts as to the ability of tariffs to make any significant difference to the scale of domestic manufacturing (Krugman 2025), while the major risks associated with global imbalances are for the financial sector rather than the manufacturing sector (Wolf 2025c).

Yet, whatever the precise underpinnings of the Trump administration’s approach to trade and international engagement, US tariff-induced trade barriers are currently at levels not witnessed since the 1920 s (Wolf 2025d), and the extent of this protectionism underpins the extent to which the global trading system is being reconfigured. Although bilateral negotiations between the US and other countries or trade blocs such as the EU are ongoing, the outcomes are uncertain and liable to change (Wolf 2025e). More fundamentally, this protectionist shift represents a profound rupture from the cross-regional transnational trade logic that has characterized the rules-based approach of the last four decades. In particular, this mercantilist trade balance logic implies profound challenges to the types of global value chains (GVCs) in which EU regions are heavily embedded (Tett 2025c). Global value chains (GVCs) represent a combination of specialization and coordination, but these traverse national boundaries, so they involve complex multinational coordination processes that do not necessarily closely align with particular national interests. Global value chains (GVCs) require the tight integration, coordination, and orchestration of supply chains and decision-making hierarchies, but four decades of modern globalization mean that modern global value chains are largely independent of nation-states, and even of many aspects of the EU Single Market. The ongoing political economy shocks to the global trade and financial systems therefore imply profound ruptures to many global value chains, constructed as they are based on numerous intertwined innovation-driven demand–supply relationships spanning sectors, regions, and countries. When a major external shock occurs, the interconnectedness between sectors can be affected (Günther et al. 2019), and this is also the case for regions. The implications of these shocks on the innovation, growth, and development trajectories can be profound, and mapping out in advance the possible economic geography of these shocks gives us a chance to design the best policy responses (Brenner and Broekel 2019).

## An empirical assessment of the implications of global protectionism on EU regional demand, supply, and global value chains

In order to examine the likely EU regional growth and innovation-related implications of mercantilist–protectionist shift in global political economy, we exploit an EU-wide interregional input–output framework which is highly disaggregated according to sectors and regions and set in the context of global trade-linkages, thereby allowing us to examine the scale of the potentially adverse impacts on EU regional economies resulting from trade wars, especially between the US and China. In particular, we use a global product–product interregional input–output table for 2017, the latest update of the *EUREGIO* global database (Thissen et al. 2018; García-Rodríguez et al. 2025; Almazán-Gomez et al. 2023, 2025). These data distinguish 64 product and services categories $p$ and all 242 NUTS2 regions of the EU27 plus the UK in a setup that is completely consistent with the FIGARO database for these countries. The year 2017 is the most recent year with the fully comprehensive data available, in part because data in subsequent years were strongly affected by disruptions due to the COVID-19 pandemic.

**Fig. 1:**
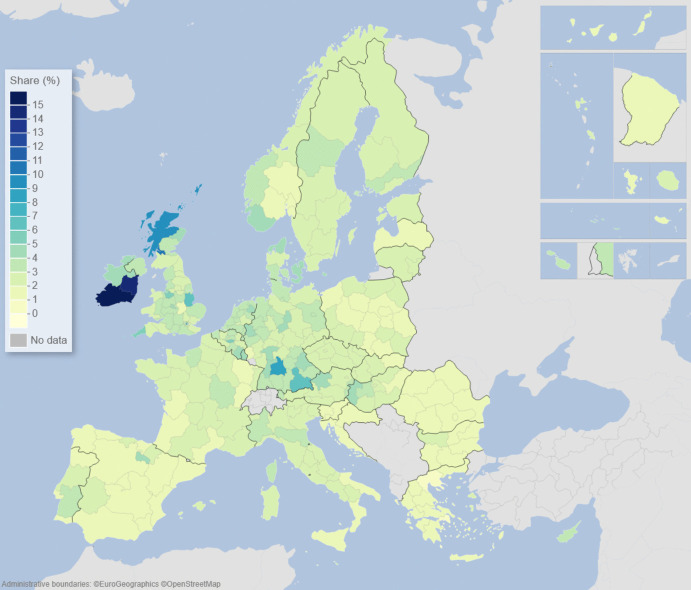
Share of European regional GDP dependent on exports to the USA

**Fig. 2:**
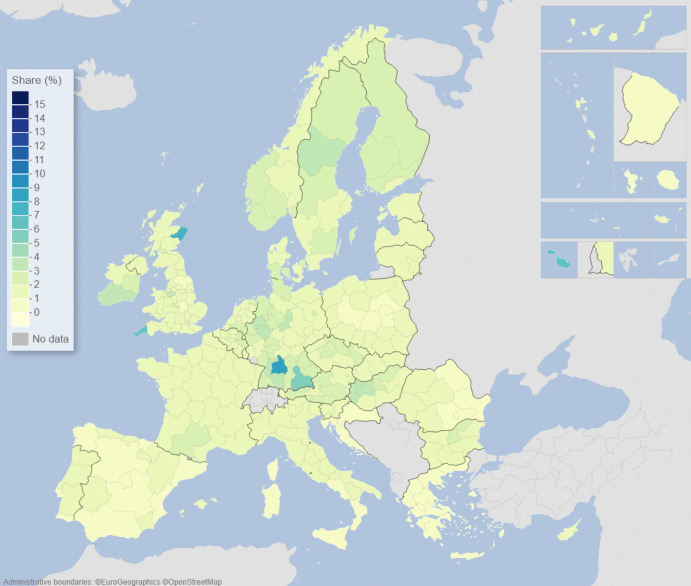
Share of European regional GDP dependent on exports to China

**Fig. 3:**
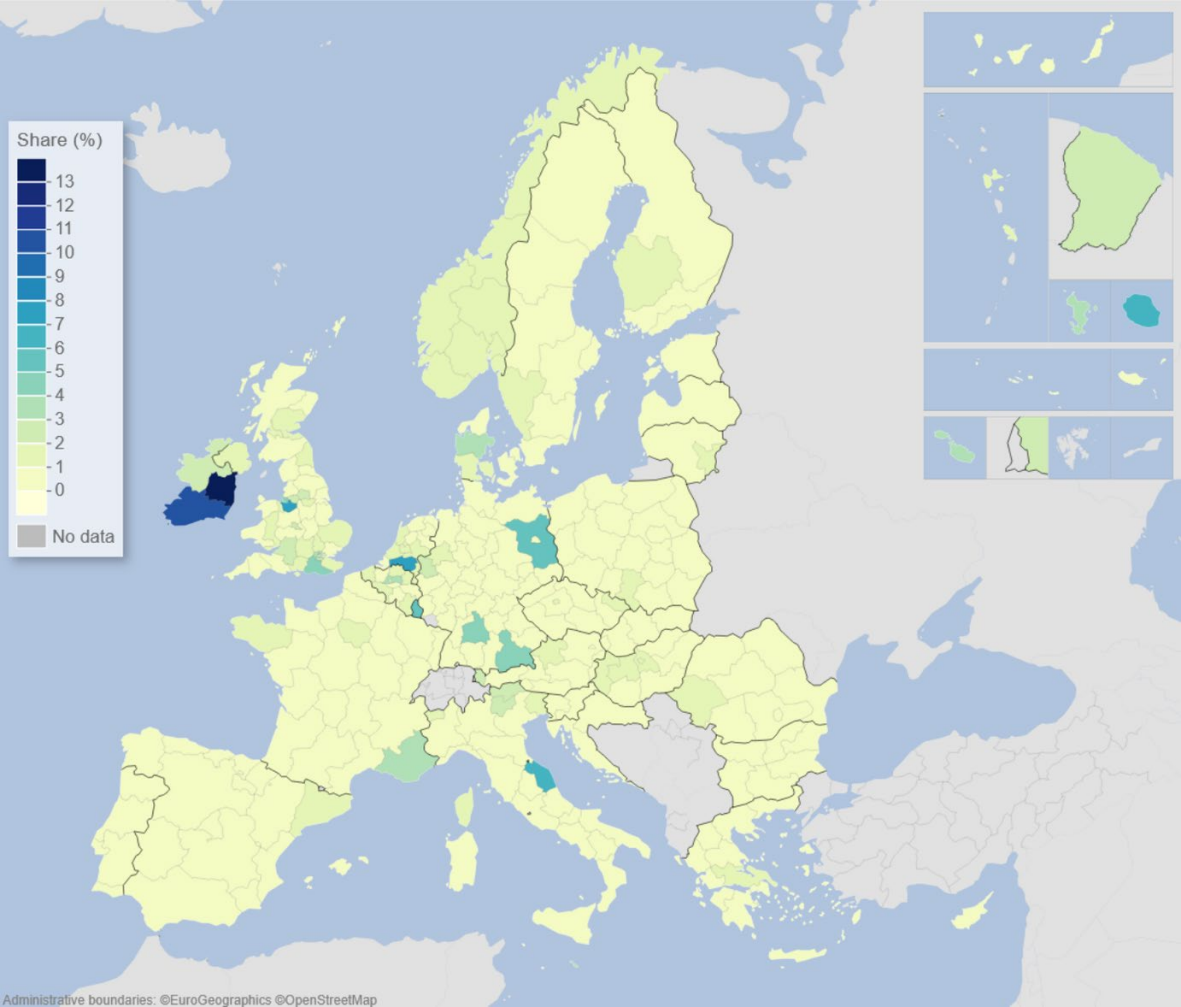
Share of European regional GDP dependent on imports from the USA

**Fig. 4:**
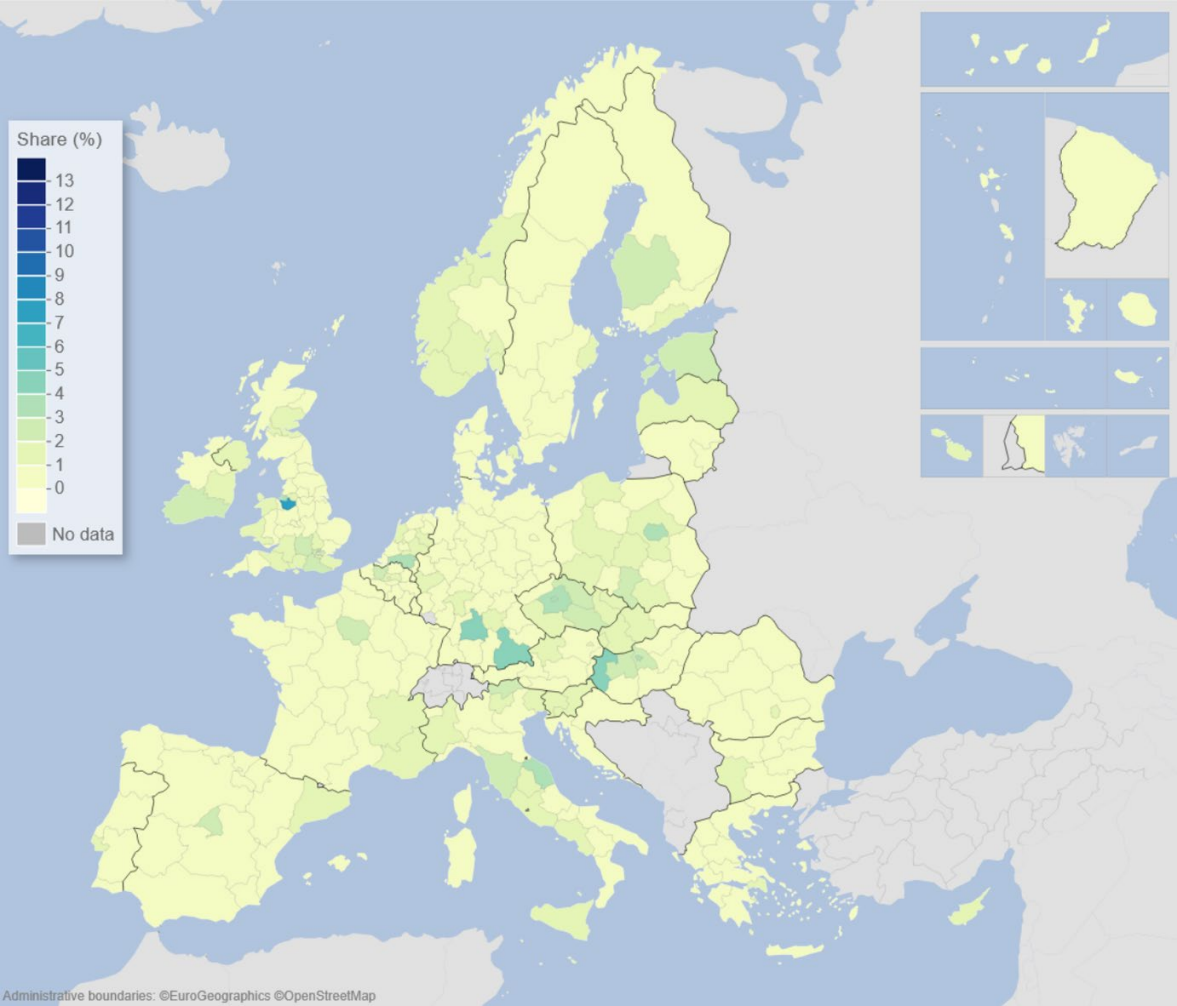
Share of European regional GDP dependent on imports from China

**Fig. 5:**
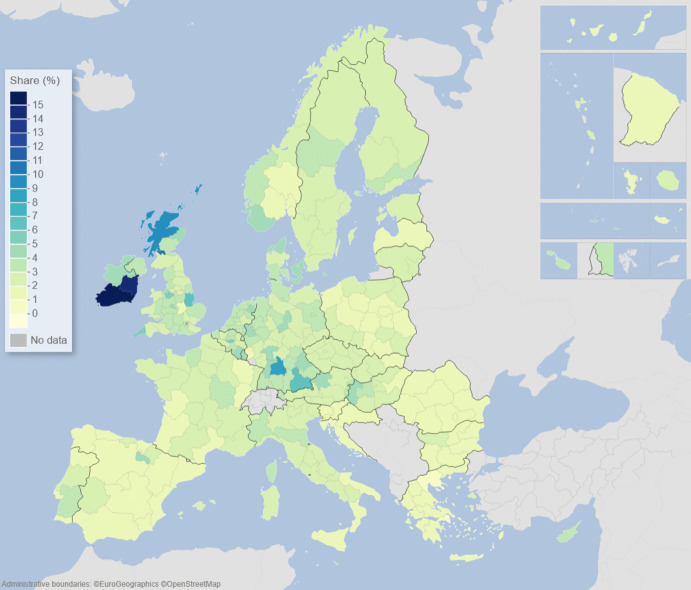
Share of European regional GDP dependent on global value chains involving the USA

**Fig. 6:**
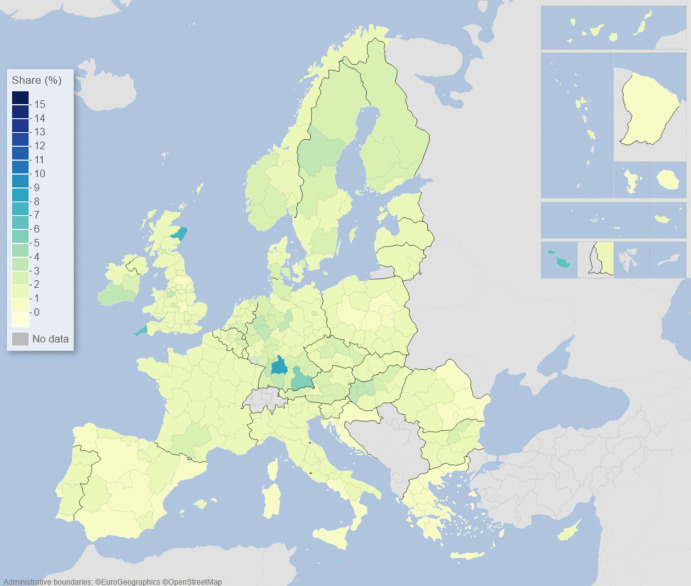
Share of European regional GDP dependent on global value chains involving China

We can describe the standard input–output model using matrix algebra as:

1 $${\boldsymbol{x}}={\boldsymbol{A}}{\boldsymbol{x}}+{\boldsymbol{f}}={\left({\boldsymbol{I}}-{\boldsymbol{A}}\right)}^{-1}{\boldsymbol{f}}$$

where ${\boldsymbol{x}}$ is a vector of region-specific and sector-specific production values, ${\boldsymbol{A}}$ is a matrix with elements describing the use of different products from different regions as a share of output value, ${\boldsymbol{f}}$ is a vector of final demand for products of different regions, and ${\boldsymbol{I}}$ is the unity matrix. The row elements of the so-called Leontief inverse ${\left({\boldsymbol{I}}-{\boldsymbol{A}}\right)}^{-1}$ represent the value share of products from a particular region needed for a demanded product in another region, represented by an element of ${\boldsymbol{f}}$. The elements of the Leontief inverse include all intermediate products used in production and therefore represent the complete value chain. Post-multiplying the production values by their region and product-specific share of GDP to production gives all value added associated with consumption in every region of the world. On the basis of this system of accounts, the respective calculated values of the demand, supply, and global value-chain impacts across EU NUTS2 regions are depicted in the maps of Figs. 1, 2, 3, 4, 5 and 6.

Figures 1 and 2 show the calculated size of the demand dependency of EU regions on the US and China, respectively, via EU exports to the USA and China. The effects on EU regional GDP are presented in Figs. 1 and 2 are derived by setting the demand in the US and China for EU imports to zero, respectively, and solving the input–output model accordingly. In Fig. 1, we see that the European regions that are most dependent on US demand are located in the Republic of Ireland, followed by southern Germany, western Hungary, Scotland, and southern Portugal, with additional noticeable impacts in various regions in France, the Low Countries, Spain, Poland, and the Baltic countries.

In terms of EU regional demand dependency on China, in Fig. 2 we see that the regions that are most dependent on Chinese demand for European exports are located in southern Germany, western Hungary, Scotland, plus parts of France, and the Low Countries. Demand dependency on China appears to be somewhat more spatially concentrated in the core of Europe than US demand dependency, which is more widespread, except for a slightly higher Chinese demand dependency in the Nordic countries.

Turning now to inputs and supplies, Fig. 3 shows the share of EU regional GDP that is dependent on imports from the USA. Here we see that parts of the Republic of Ireland, Italy, the Low Countries, and southern and eastern Germany are the most dependent on imports from the USA. In contrast, as we see in Fig. 4, the EU regional dependency on imports from China is most noticeable in southern Germany, western Hungary, the Czech Republic, Poland, and other parts of Central Europe.

If we now combine both demand and supply aspects of engagement with the US and China, along with all of the myriad intermediary roles played by third-party countries outside of Europe via global value chains (GVCs), we are able to construct the overall GVC dependency of EU regions on the USA and China. What is clearly observable when comparing Figs. 5 and 6 is that the GDP of European regions in general is more dependent on global value chains (GVCs) driven by engagement with the USA rather than those connected with or via China. In Fig. 5, we see that these GVC dependencies on the USA are most marked in the Republic of Ireland, Scotland, central and southern Germany, the Low Countries, plus parts of western Hungary and Portugal. In Fig. 6, we see that the GVC dependencies on China are most marked in central and southern Germany, Scotland, and western Hungary. The dependence of EU regional GDP on GVCs engaging with the USA is widely spread across the EU and typically with higher levels of dependency across more regions than is the case with engagement with China, whose GVC-related impacts tend to be more concentrated in the center of Europe.

Figures 1, 2, 3, 4, 5 and 6 depict, in effect, the level of risk exposure that each region faces due to the imposition of tariffs and trade wars. Depending on precisely where in the international demand arenas, supply arenas, and global value chains (GVCs) that each region is situated, the potential impacts of any trade export, import, or GVC disruption differ markedly. European regions with greater dependencies on these external demand, supply, and global value chains face greater exposure risks associated with international connectedness. However, the specific adverse GDP impacts on each European region cannot be known in advance, because they depend not only on the scale of any tariff-induced demand shocks but also on the particular origin of the trade disruption. Global geo-economic trade fragmentation significantly increases the complexity and unpredictability of the overall economic environment (ECB 2024), and we need to consider the likely regional implications of this extra layer of uncertainty to the whole question of regional risk exposure.

**Fig. 7:**
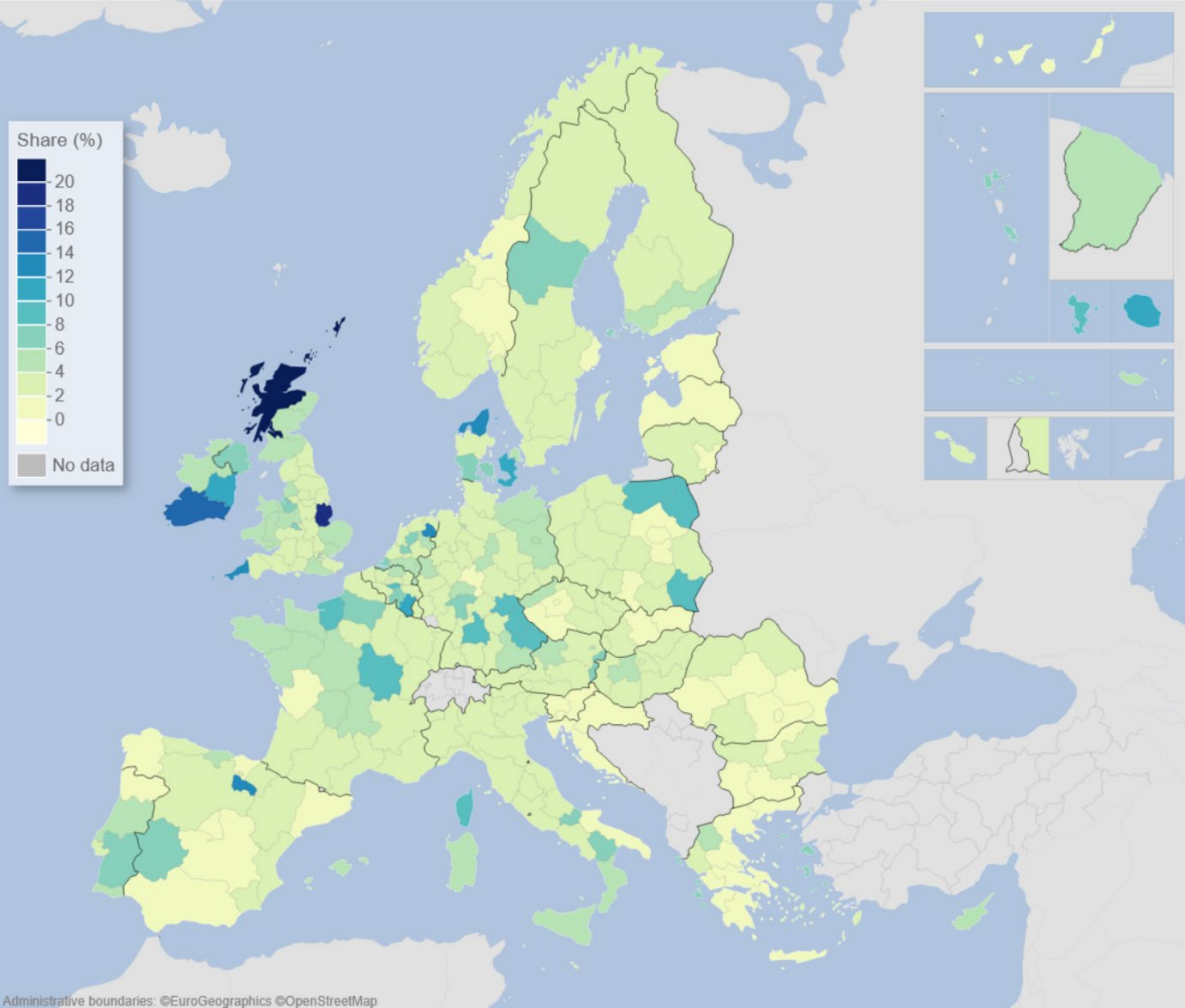
Standard deviation of European regional GDP dependent on global value chains involving the USA

**Fig. 8:**
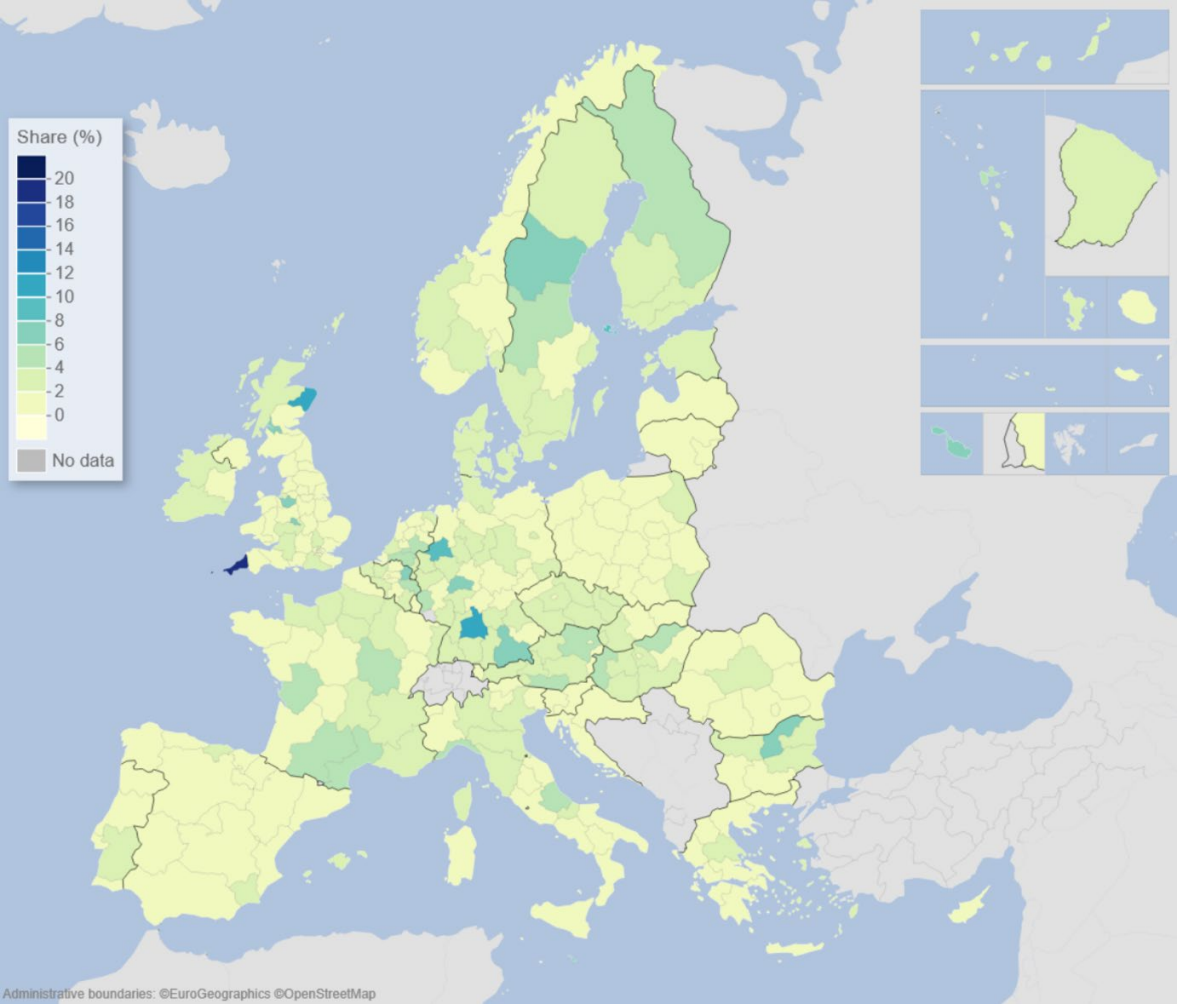
Standard deviation of European regional GDP dependent on global value chains involving China

Using the same empirical system, we are able to examine the scale of this uncertainty. In order to do this, we can examine the range of impacts from trade shocks on each of the individual sectors present in the region and assess their dispersion. The results for the origin and destination regions were taken directly from the IO tables. The weighted standard deviation $\sigma$ of sector results for every region is then calculated from the sector results as follows:

2 $$\sigma =\sqrt{{\sum }_{p}{{\omega }_{p}\left({y}_{p}-\overline{y }\right)}^{2}}$$

where ${\omega }_{p}$ is the weight of this product in the total regional GDP, ${y}_{p}$ is the product-specific effect via the global value chain (GVC) in percentages of a reduction in demand from the USA and China, respectively, and $\overline{y }$ is the weighted average of the effect in percentages as presented in Figs. 5 and 6. The calculated values provide an index of the uncertainty associated with each region’s trade-related risk exposure. The results of this exercise are depicted in Figs. 7 and 8.

**Fig. 9:**
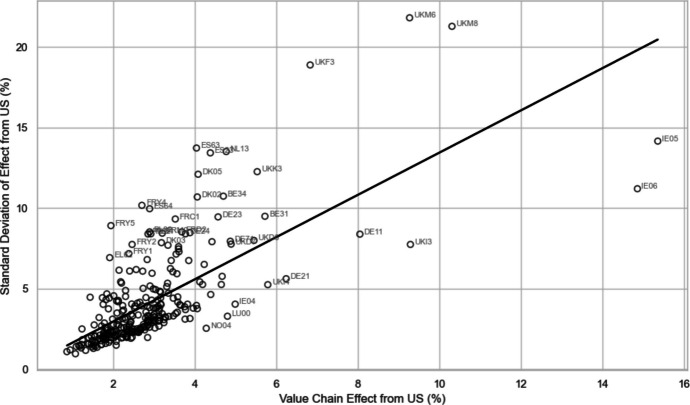
Relationship between European regional uncertainty (standard deviation) and level of economic risk exposure (level) of value-chain effect with the USA (Pearson correlation coefficient = 0.698)

**Fig. 10:**
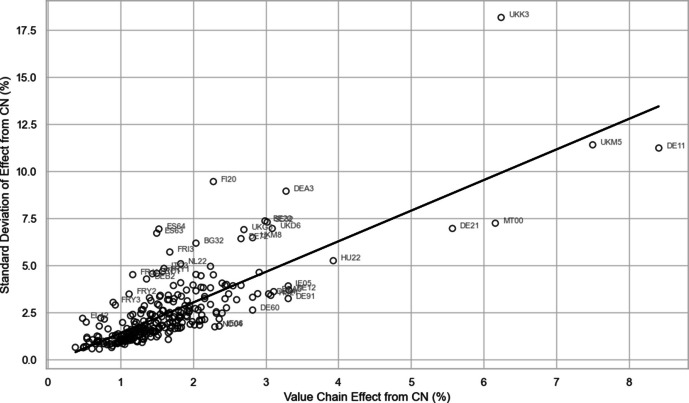
Relationship between European regional uncertainty (standard deviation) and level of economic risk exposure (level) of value-chain effect with China (Pearson correlation coefficient = 0.787)

In Fig. 7, we see that the levels of exposure risk uncertainty are both greater and also across a wider range of regions for trade with the USA than is the case with China. The uncertainty related to potential USA trade shocks is spread widely across European regions, including those located in the northern and eastern flanks of the EU, as well as many southern European regions. As such, both the overall USA-related economic exposure risks and the allied uncertainty associated with these risks are relatively high and widely spread across Europe, but the uncertainty is more widely felt, even including in more peripheral regions. In comparison, for China-related trade, both the economic exposure risk and the additional uncertainty associated with these risks tend to be both lower and also more geographically concentrated in the center of Europe. As we see in Figs. 9 and 10, the European regional correlation between the uncertainty of GVC-related exposure risks and the level of GVC-related economic exposure risks is 0.698 for trade and engagement with the USA, and 0.787 for trade and engagement with China. In other words, regions with greater levels of tariff- or protectionist-induced economic risk exposure also typically face higher additional uncertainty about the levels of economic risk they are likely to face. The problems are more significant and widespread for US-related risks, whereas China-related risks are more concentrated in regions at the economic center of Europe.

In order to assess what these trade and global value chain (GVC) related exposure risks and uncertainty imply for different types of European regions based on their regional innovation characteristics, following Prenzel et al. (2018), we employ the OECD innovation classification (OECD 2011b; Marsan and Maguire 2011) of European regions set at the OECD-TL2 levels^2^. This classification assigns each EU region into one of seven different innovation types, namely: traditional manufacturing regions (DUI); knowledge-intensive city/capital districts (STI); medium tech manufacturing and service providers (DUI); knowledge and technology hubs (STI); structural inertia or deindustrializing regions (DUI); service and natural resource regions in knowledge-intensive countries (DUI); primary-sector-intensive-regions (DUI).

All types of regions will comprise firms whose innovation dynamics are either STI-driven, DUI-driven, or even combinations of these two, and therefore when describing these seven types of innovation-characteristics regions in terms of their overall STI or DUI features, as classified by the OECD (Marsan and Maguire 2011; OECD 2011b), we consider what are likely to be the dominant modes of innovation prevalent in the region. We therefore ascribe these seven different regional innovation types into STI-driven or DUI-driven regions, and in this classification system, for the purposes of this paper, only knowledge-intensive city/capital districts and knowledge and technology hubs would be classified as STI-innovation regions. All other regions we ascribe to the DUI-driven innovation regions classification. Regions arrive at displaying these different innovation-features as a result typically of many years of path dependence, regional lock-in, and co-evolution, and the specific evolutionary pathways traversed by different regions will differ, and in some cases will be unique. Our empirical analysis does not allow us to examine these region-specific developmental pathways, but it does allow us to examine the likely trade exposure risks and uncertainty by regional type.

**Fig. 11:**
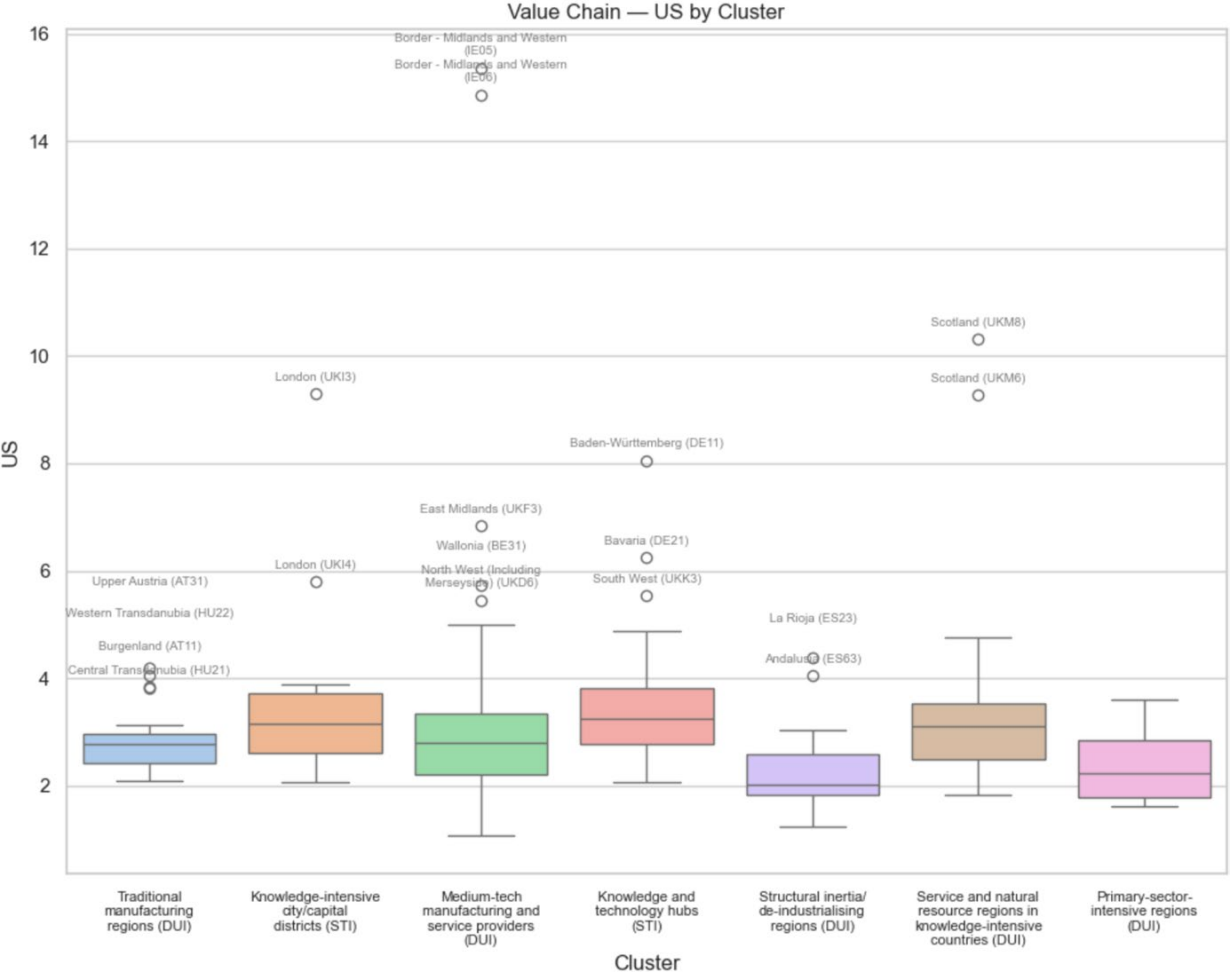
Share of European regional GDP dependent on global value chains involving the USA by regional innovation type

**Fig. 12:**
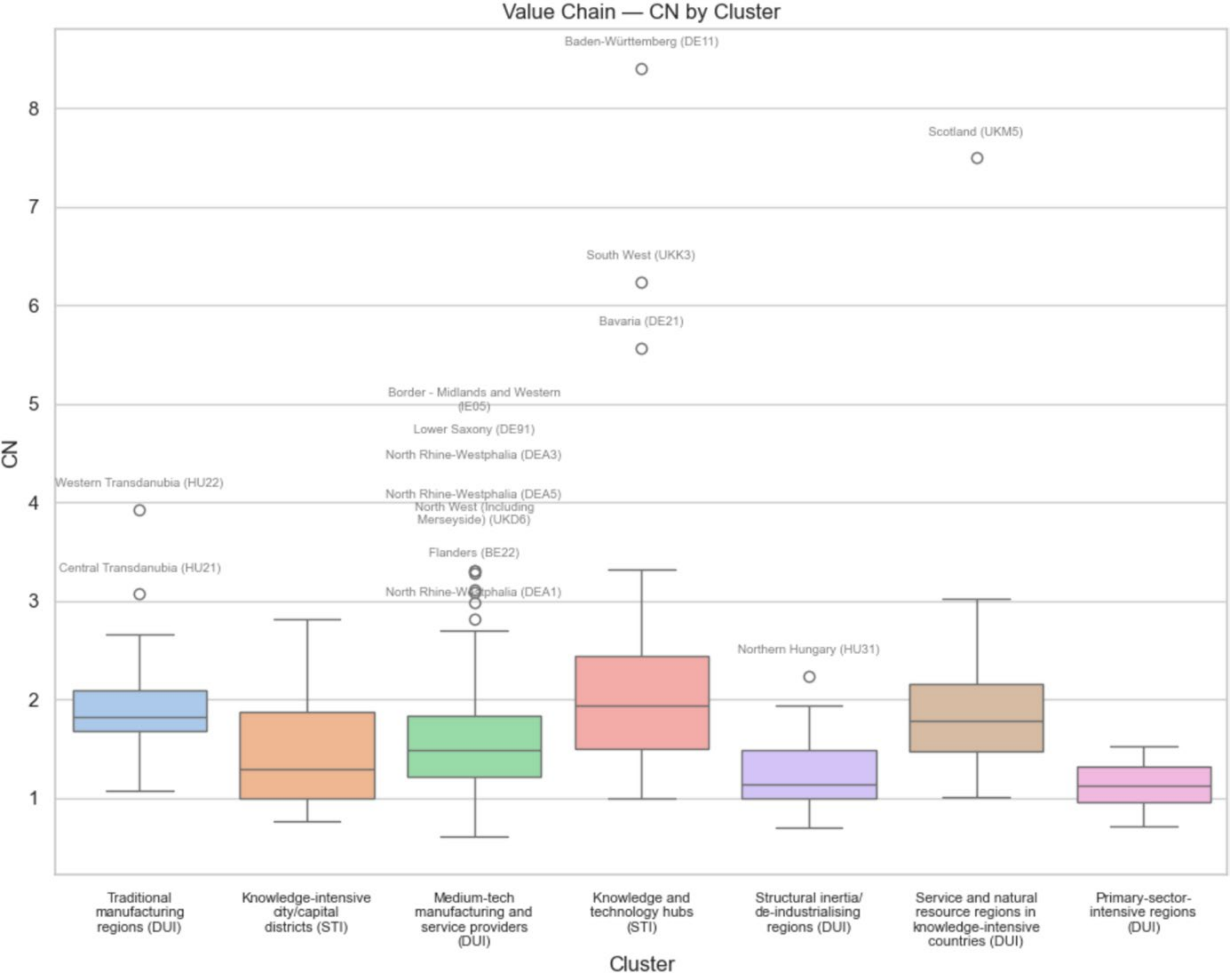
Share of European regional GDP dependent on global value chains involving China by regional innovation type

**Fig. 13:**
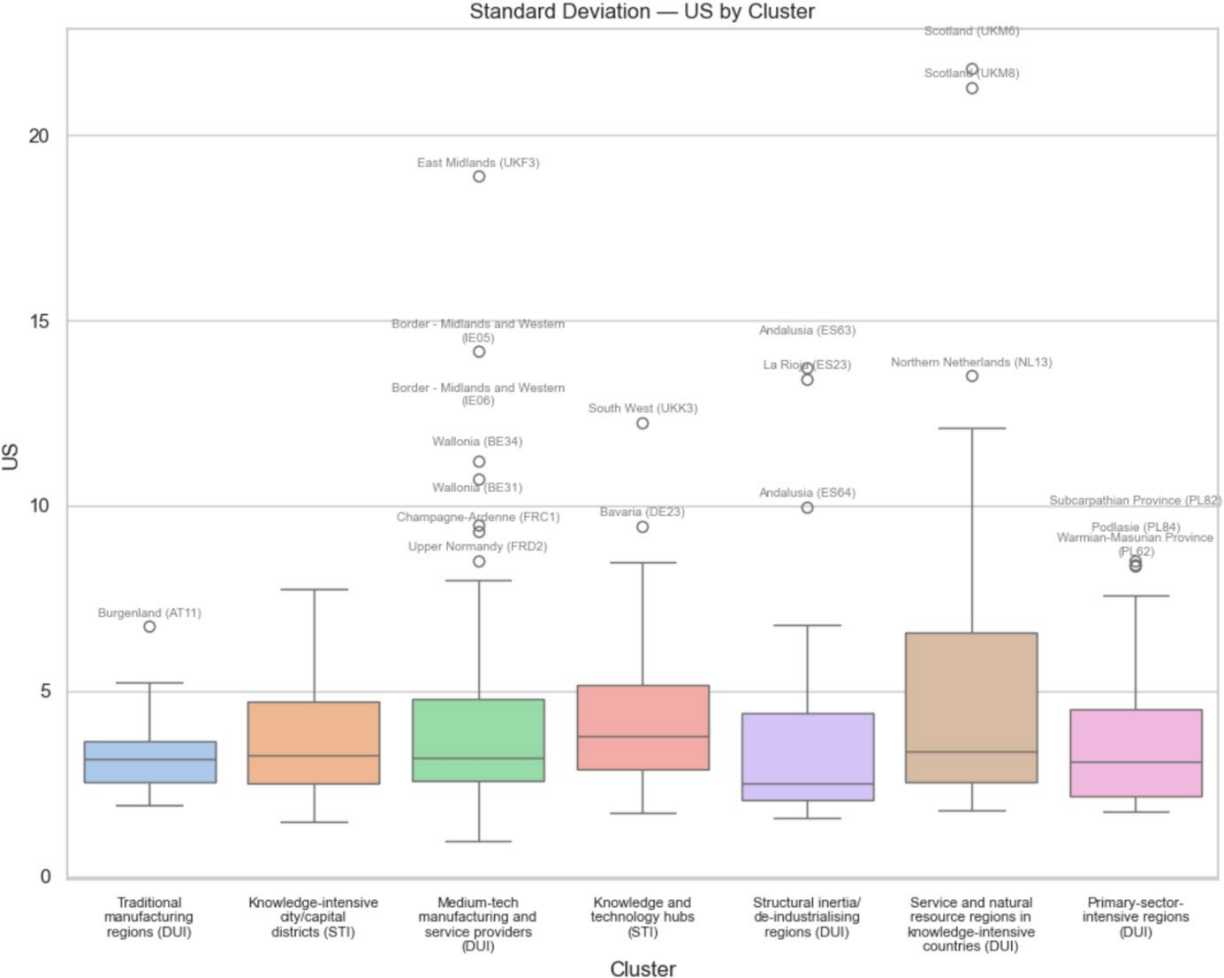
Standard deviation of European regional GDP dependent on global value chains involving the USA by regional innovation type

**Fig. 14:**
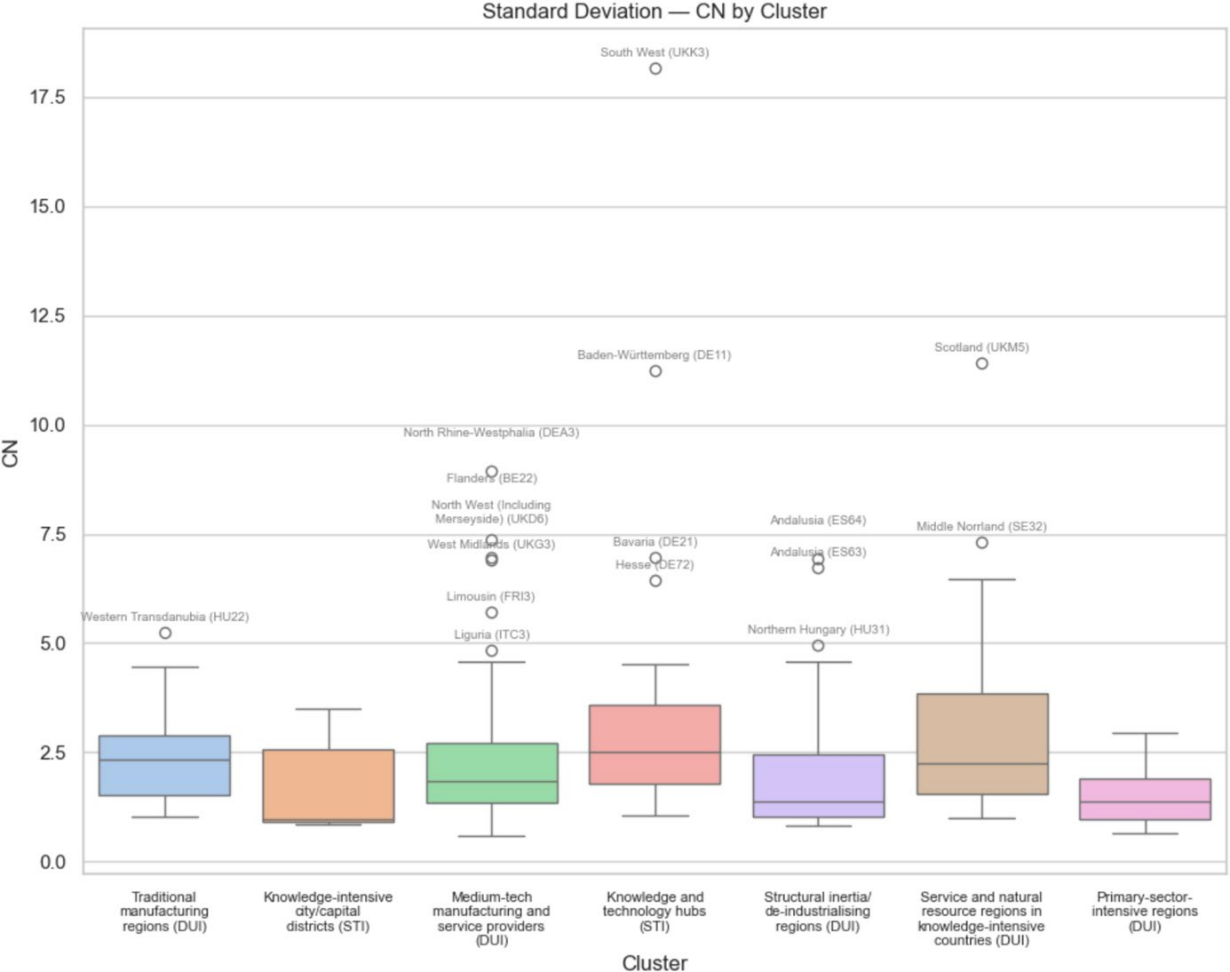
Standard deviation of European regional GDP dependent on global value chains involving China by regional innovation type

Figures 11, 12, 13 and 14 show the box plots for each of the seven innovation types of regions broken down according to the quartiles of the respective distributions, plus the extreme outliers of each category. Figure 11 shows the share of European regional GDP that is dependent on GVCs involving the US according to the seven regional innovation classification groupings, and Fig. 12 does this with respect to GVCs linking EU regions to China. Figures 13 and 14 show the respective box plots according to the seven innovation groupings for regional uncertainty based on the standard deviation of outcomes.

Comparing Figs. 11 and 12, we see that the median regional risk exposure for all regional innovation types with respect to the USA is typically about double that of the risk exposure with respect to China. Similarly, comparing Figs. 13 and 14, we see that the median uncertainty associated with the standard deviation of regional risk exposure for all regional innovation types with respect to the USA is typically about double that of the risk exposure to China.

In terms of risk exposure to increasingly restricted US markets, we see in Fig. 11 that while knowledge and technology hubs and knowledge-intensive city/capital districts are the two most exposed types of regions, both medium tech manufacturing and service providers, along with service and natural resource regions in knowledge-intensive countries, are also relatively highly exposed, as are traditional manufacturing regions. Structural inertia or deindustrializing regions, and primary-sector-intensive-regions are relatively less exposed. In contrast, in terms of trade restrictions into Chinese markets, we see in Fig. 12 that after the knowledge and technology hubs, the next most exposed regions are the traditional manufacturing regions plus the service and natural resource regions in knowledge-intensive countries, followed by medium-tech manufacturing and service providers. Knowledge-intensive city/capital districts face relatively low exposure. The least innovative regions, namely Structural Inertia or deindustrializing regions and primary-sector-intensive-regions face the lowest exposure risk.

When we consider the levels of uncertainty of the risk exposure, associated with the standard deviation of regional risk exposure, we see in Fig. 13 that along with knowledge and technology hubs, it is the medium-tech manufacturing and service providers and the service and natural resource regions in knowledge-intensive countries that face the most uncertainty with regard to the USA. For uncertainty associated with trade with China, we see in Fig. 14 that it is these same three types of regions, plus also the traditional manufacturing regions, that face the most uncertainty. Again, the least innovative regions, namely structural inertia or deindustrializing regions and primary-sector-intensive-regions face the lowest uncertainty.

On the other hand, when we consider the EU outlier regions facing exceptionally high trade exposure risks and uncertainty, we see from Figs. 11, 12, 13 and 14 that all of the different regional innovation types feature, except for the knowledge-intensive city/capital districts. Allied with the fact that the overall risk and uncertainty median and mean values for knowledge-intensive city/capital districts are always relatively low, except for trade exposure to the USA, this implies that it is other types of regions, including many less innovative regions, that face some of the greatest challenges from trade disruption. As such, taken together, our results suggest that the mercantilism-driven trade exposure risks and uncertainty affect all of the different types of regional innovation categories. In particular, there is no simple STI-driven versus non-STI/DUI-driven regional innovation pattern to these trade-related risks, nor is there any simple leading-versus-lagging/core-periphery pattern to these risks and uncertainty. It depends on where regions are positioned within specific global value chains (GVCs).

## Discussion: Lagging regions and lagging economic narratives

The importance of our analysis lies in the fact that although we know that evolutionary economic processes are always characterized by risk and uncertainty (Lehmann-Waffenschmidt and Peneder 2022), it is still important to try to calibrate the levels of risk and degrees of uncertainty in order to build an overall picture of the likely evolutionary growth processes in response to specific shocks. International trade and endogenous technological change are closely interrelated phenomena (Antonelli and Feder 2021).

Innovation potential and global value chains are intrinsically linked (Ito et al. 2023; Bontadini et al. 2024; EIB 2025), and uncertainty is the largest barrier to investment in Europe (EIB 2025). Evidence regarding the likely differential European regional implications of global trade shocks and trade wars is therefore essential in order to consider the economic geography of innovation and growth, and also the likely political and civil responses to both the shocks and to any EU policy responses. Part of the problem here, however, is that regarding the efficacy of any policy responses, in terms of economic geography, much of the most influential latest thinking in the highest echelons of the European institutions is still based on framings which are more than 15 years out-of-date from today’s realities of economic growth.

A key example is the case of the two recent and highly influential reports by Mario Draghi (Draghi 2024a, b) and Enrico Letta (Letta 2024), which focused on the need for urgent and profound structural changes to the European economy as a whole (Draghi 2024a, b) and also to the workings of the EU Single market (Letta 2024), in order to galvanize Europe’s growth. Letta (2024) focused on removing ongoing barriers to integration within the EU Single Market. Meanwhile, Draghi emphasized the need for a re-prioritization of reforms aimed at re-invigorating Europe’s ‘growth engine’ with a focus on changes in the policy approaches of both the European Commission and the national governments of member states relating to trade, energy, and defense, including a much deeper integration of Europe’s capital markets. Curiously, however, while both reports acknowledge that Europe’s overall growth rate had declined in recent years, neither report considered at all how the dynamics of economic growth within Europe and the EU Single Market had also fundamentally changed. Both reports were still based on the 1980 s and 1990 s growth and convergence framings in which asking questions regarding regional distributional aspects was deemed unnecessary, because the requisite knowledge diffusion and dissemination processes were assumed to operate throughout the market, facilitated by the removal of intra-EU barriers to integration. However, in marked contrast today, however, widespread regional divergence means that asking such questions is crucial in order to understand which policy approaches and settings might be workable, as we now briefly explain.

From the post-war years of the 20th century onwards, all Western European economies, and also Western Europe as a whole, enjoyed growth processes driven by conditional convergence (Carrascal-Incera et al. 2020), and this was the analytical framework underpinning the construction of the EU Single Market (McCann 2015). In the immediate aftermath of the global financial crisis, however, it was becoming clear to increasing numbers of European (Barca et al. 2012) and OECD-wide (OECD 2011a) observers that the previous convergence-based analytical and empirical framings were already out of date in the European context. Most industrialized economies today experience economic divergence, not convergence between their regions (Garcilazo and McCann 2026), and this was first documented in the EU employment context in 2014 (European Commission 2014). While there is still some ongoing growth convergence between EU13 and EU15 countries, this has also slowed markedly. In other words, the knowledge diffusion and dissemination processes assumed to be inherent in the EU Single market appear not be playing the growth-convergence roles that they were assumed to do. In terms of the geography of EU innovation, this declining connectivity is a crucial weakness, and the fact that much of Europe has shifted from a high-growth-convergence regime to a slow-growth-divergence regime suggests that greater competition and integration are, of themselves, unlikely to provide a solution to these challenges. A deeper understanding of how the different parts of Europe are connected or not would appear to be essential, and this underscores the importance of the trade-shocks and global value-chain analyses reported earlier.

Yet, although much of the pre-crisis convergence-based thinking applied to the post-crisis was already out-of-date within a few years of the aftermath of the 2008 crisis, the high-level convergence-based framings were barely challenged for many years after the crisis because of the slow emergence of countervailing evidence as well as high-level EU institutional attention being focused elsewhere on the Euro crisis, then Brexit, and then later again, COVID-19. It was only in 2011 and 2012 that consistent and standardized OECD-NUTS datasets were first produced for both European regions and cities and it was not until 2015 with the advent of the EU RHOMOLO model^3^, the EU’s computable general equilibrium model (Brandsma et al. 2015), along with the 2018 *EUREGIO* regional extensions to the EU input-output model consistent with the FIGARO datasets (Thissen et al. 2018; García-Rodríguez et al. 2025), that empirical evidence confirmed that the EU’s economic geography of growth had indeed changed dramatically in the post-crisis period from what was typically observed in the pre-crisis era. The common patterns in capital market shocks across the USA (Daams et al. 2024a), the UK (Daams et al. 2024b), and the rest of Europe (Daams et al. 2025a, b) strongly suggest that these convergence-divergence regime switches were driven by global financial market dynamics rather than by specific public policies. Yet, the lessons arising from these shocks have still not permeated many of the higher echelons of EU policy thinking, presumably because the attentions of EU institutions were first focused on the Eurocrisis, then diverted from 2016 onwards by Brexit, followed by the COVID-19 crisis.

The fact that neither Draghi nor Letta even mentions any of these issues underscores the extent to which high-level thinking in Europe is still operating in a ‘space-blind’ framing of the pre-crisis years in which growth and regional convergence are just assumed to be inherently co-determined (Barca et al. 2012). They are not, and across Europe, they have not been so for over 15 years. Neither Draghi (2024a, b) nor Letta (2024) has caught up with these realities McCann and Stierna 2025), nor has much of the mission-oriented thinking also emerging from Brussels (Janssen et al. 2024). Across European regions, knowledge diffusion and dissemination no longer take place automatically as a natural corollary of the removal of internal trade barriers and competition processes, and the European economic geography of innovation is not becoming less uneven and more efficient, but more unbalanced and geographically concentrated. Therefore, assessing how the geography of likely trade shocks and global value chains (GVCs) interacts with other EU energy (McCann and Soete 2020; OECD 2023), defense and policy priorities, and in turn how these may collectively impact on modern EU innovation policies (McCann and Ortega-Argilés 2015), will help us to better understand the evolving EU regional innovation landscape.

## Conclusion

Trade protectionism, mercantilism, and trade wars are becoming the norm (The Economist 2025a, b: Sandbu 2025). As we demonstrate in this paper, all these extra layers of trade-related risk exposure and uncertainty add further complexity to the EU geography of innovation. Local innovation opportunities and financing depend crucially on the structure and buoyancy of the local economy, and when regions are hit by external shocks, the local innovation system may be weakened. Many of these mercantilist shocks cannot be predicted on the basis of the current European economic geography of innovation, but are a result of political choices and the positioning of specific regions in global value chains (GVCs). Our analyses using the *EUREGIO* datasets allow us to understand the combination of direct demand-transmission effects and the indirect effects of GVCs spanning EU regions and non-EU countries.

As we demonstrate, the levels of economic exposure-risks faced by different innovation-types of EU regions to mercantilist-led trade protectionist shocks differ between places. In general, trade shocks originating from both the USA and China are more likely to adversely impact central and northern urbanized regions of Europe rather than more peripheral and economically weaker regions, with larger overall effects associated with the USA but more spatially concentrated effects associated with China. At the same time, while many STI-driven knowledge and technology hubs face high levels of trade-related risk and uncertainty, this is also the case for many non-STI/DUI-driven regions, such as medium-tech manufacturing and service providers, traditional manufacturing regions, plus the service and natural resource regions in knowledge-intensive countries. Indeed, although the average exposure risks and uncertainty are typically low for structural inertia or deindustrializing regions and primary-sector-intensive-regions, even some of these types of regions face very significant trade-related risks and uncertainty. On the other hand, apart from risk exposure to the USA, in general, the trade-related risk and uncertainties faced by knowledge-intensive city/capital districts are actually lower than for most other types of regions. As such, if, as part of the effort to boost Europe’s open strategic autonomy (Fontagné and Yotov 2024), the types of policies put forward by Draghi (2024a, b), Letta (2024), and the mission-oriented agenda (Janssen et al. 2024) bolster the relative positions of knowledge-intensive city/capital districts, they are likely to exacerbate EU regional inequalities. Yet, any European attempts at decoupling from global value chains (GVCs) are unlikely to be helpful (Eppinger et al. 2021). Rather, a careful analysis of the global value chain (GVCs) implications in which specific EU regions are embedded is essential not only for understanding the likely regional innovation evolutions, but also for tailoring policy to best respond to these shocks.

## Data Availability

The EUREGIO datasets are publicly available

## Footnotes

1 https://eu.eventscloud.com/website/4590/home/

2 For much of Europe, EU-NUTS2 regions coincide with OECD-TL2 regions, but where OECD-TL2 regions coincide with EU-NUTS1 regions, we classify each EU-NUTS2 region as the same innovation category as the respective EU-NUTS1 region in which they are nested (Prenzel et al. 2018).

3 https://joint-research-centre.ec.europa.eu/projects-and-activities/territorial-data-analysis-and-modelling-tedam/regional-holistic-model-rhomolo_en
